# Perme ICU Mobility Score (Perme Score) and the ICU Mobility Scale (IMS): translation and cultural adaptation for the Spanish language

**DOI:** 10.25100/cm.v49i3.4042

**Published:** 2018-12-30

**Authors:** Esther C Wilches Luna, Nasly L Hernández, Anamaria Siriani de Oliveira, Ricardo Kenji Nawa, Christiane Perme, Ada Clarice Gastaldi

**Affiliations:** 1 Grupo de Investigación Ejercicio y Salud Cardiopulmonar (GIESC), Cali, Colombia.; 2 Universidad del Valle. Facultad de Salud Escuela de Rehabilitación Humana. Cali, Colombia.; 3 Clínica Farallones, Unidad de Cuidado Intensivo Adultos, Christus Sinergia. Sociedad de Fisioterapeutas Respiratorios (SOFIRE SAS).Cali, Colombia.; 4 Universidade de São Paulo, Departamento de Ciências da Saúde, Programa de Pósgraduação em Reabilitação e Desempenho Funcional. Ribeirão Preto, Brasil; 5 Hospital Israelita Albert Einstein São Paulo , Brasil; 6 Houston Methodist Hospital, Department Rehabilitation Services, Board Certified Cardiovascular and Pulmonary Clinical Specialist by the American Physical Therapy Association. Houston, United States; 7 Universidade de São Paulo, Departamento de Ciências da Saúde, Laboratório de Avaliação Respiratória. Ribeirão Preto, SP, Brasil​

**Keywords:** Critical illness, early ambulation, length of stay, respiration, artificial, language, muscle weakness, physical therapists, critical care, Perme Intensive Care Unit Mobility Score, The ICU Mobility Scale (IMS), Enfermedad critica, movilidad temprana, duración de la estancia, respiración artificial, lenguaje, debilidad muscular, terapia física, cuidado critico, Perme Intensive Care Unit Mobility Score, The ICU Mobility Scale (IMS)

## Abstract

**Introduction:**

The scales to measure functional mobility in critically ill patients were developed and validated in English, there is a need for these tools in Spanish speaking countries.

**Objective:**

To perform translation, cultural adaptation and inter-rater reliability of the Spanish versions of the Perme Intensive Care Unit Mobility Score and IMS tools in ICU patients.

**Methods:**

Translation and validation study between November 2016 and July 2017, following the COSMIN Protocol's recommendations. Two couples of physiotherapists with the role of observer/rater applied both scales in 150 patients upon admission and discharge of a medical-surgical ICU from a private hospital in Colombia. The sample size was defined taking into account the lowest proportion of reported agreement (68.57%), a Kappa index of 0.2784 or higher to ensure that the calculated n was adequate, and a confidence level of 95%

**Results:**

Translation and cultural adaptation were performed, the final version of both scales in Spanish was approved by the authors. The sample was 150 patients, 52% were men, the average age was 58 ± 17 years, invasive mechanical ventilation was present in 63 (42%) of the cases. Inter-rater reliability of the ICU Mobility Scale was between 0.97 and 1.00, and for the Perme Intensive Care Unit the Mobility Score it was between 0.99 and 1 in the two moments of the measurements.

**Conclusions:**

Both scales were translated and culturally adapted and presented excellent inter-rater reliability in the two pairs of raters (rater/observer).

## Resumen

**Introducción:**

Las escalas para medir la movilidad funcional en el paciente crítico han sido desarrolladas y validadas en lengua inglesa, existe una necesidad de contar con estas escalas en nuestros países hispanoparlantes.

**Objetivo:**

Realizar traducción, adaptación cultural y determinar confiabilidad inter evaluador de la versión en español del Perme Intensive Care Unit Mobility Score y del ICU Mobility Scale (IMS).

**Métodos:**

Estudio de traducción y validación entre noviembre de 2016 y Julio de 2017 siguiendo las recomendaciones del Protocolo COSMIN. Dos parejas de fisioterapeutas con el rol de observador/evaluador aplicaron ambas escalas en 150 pacientes al ingreso y egreso de una UCI médico-quirúrgica de una clínica privada en Colombia. Se definió el tamaño de muestra teniendo en cuenta la menor proporción de concordancia reportada (68.6%), un índice Kappa 0.2784 o superior para garantizar que el n calculado fuera adecuado, y un nivel de confianza de 95%

**Resultados:**

Se realizó la traducción y adaptación cultural, la versión final de ambas escalas en idioma español fue aprobada por los autores. La muestra fue de 150 pacientes, 52% fueron hombres, la edad promedio fue de 58 ±17 años, la ventilación mecánica invasiva estuvo presente en 63 (42.0%) de los casos. Se encontró confiabilidad inter-evaluador del ICU Mobility Scale entre 0.97 y 1 y para Perme Intensive Care Unit Mobility Score estuvo entre 0.99 y 1.00 en los dos momentos de mediciones.

**Conclusiones:**

Ambas escalas fueron traducidas y adaptadas culturalmente y presentaron excelente confiabilidad inter-evaluador en las dos parejas de evaluadores (evaluador/observador).

## Introduction

Early mobilization in intensive care units (ICUs) is a strategy to improve functional recovery during and after prolonged critical illness, decrease ICU-acquired muscle weakness (ICU AMW), delirium, and also to reduce hospital stay ^1^ ^-^ ^3^

The outcome measures described in the research on mobility in the critically ill patient were the length of ICU and hospital stay, the duration of mechanical ventilation, muscle strength, functionality and mortality ^4^ ^,^ ^5^ . However, none of these results constitute specific and sensitive assessments of the patient's mobility situation. 

A total of 26 instruments to measure the function of critical patients was identified in a systematic review ^6^; however, only six instruments were specifically designed to measure functional status in critical patients with a mobility-related concept, such as: Physical Function in Intensive Care Test scored (PFIT-s), Functional *Status Score for the ICU* (FSS-ICU), Chelsea Critical Care Physical Assessment Tool (CPAx), ICU Mobility Scale (IMS), Perme *Intensive Care Unit Mobility Score (Perme Score),* Surgical Intensive Care Unit Optimal Mobility Scale (SOMS), and they were originally designed in English ^7^ ^-^ ^13^ .

Both the IMS and the *Perme Score* have demonstrated adequate levels of reliability when applied to diverse patient populations hospitalized in the ICU. Translation and cultural adaptation studies have been carried out in Portuguese and German over the last two years ^14^ ^,^ ^15^. 

Given the increasing use of Early Mobilization (EM) in ICU patients ^9^ ^,^ ^10^ ^,^ ^13^, there is a need in Spanish-speaking countries to have culturally valid instruments to longitudinally measure the ICU patient's physical function. To date, there are no known studies in our region that describe the functional mobility status of patients upon discharge from the ICU, probably due to the lack of reliable and valid tools to assess the mobility status during critical illness.

The use of an instrument that already exists in another country's a language and culture, must go through a cultural adaptation and validation process before being implemented in order to verify if the new version is reproducible and reliable ^6^.

Having instruments to measure patient mobility will improve the early identification of deficiencies, allow for earlier intervention, and the provision of services that reduce the impact of hospitalization on the patient's functional independence and quality of life. The aim of the study was to carry out the translation and cultural adaptation, and to measure inter-rater reliability of the Spanish version of the Perme Score and the IMS.

## Materials and Methods

### Patients and Methods

We conducted a translation and validation study following the COSMIN checklist's criteria (Consensus-based Standards for the Selection of Health Measurement Instruments) ^16^. The authors of the original version of the two scales authorized the process. This study was approved by the Human Ethics Committee of Universidad del Valle and the Committee of the Institution (002-005) where the research was conducted in the period from November 2016 to July 2017

### Scales Description

The "Perme Intensive Care Unit Mobility Score" (Perme Score), has good inter-rater reliability (ICC: 0.98801) (13), and was translated and validated into Portuguese in ICU patients (inter-rater reliability (κ: >0.9 e: >0.9 for most of the domains). It contains 15 items grouped into 7 categories: mental state, potential barriers to mobility, functional strength, bed mobility, transfers, walking and resistance. The score varies from 0 to 32; uses a maximum range of 2 to 4 points for each of the 15 items included. A high total score indicates few barriers to mobility and minimal assistance required for mobility activities ^11^.

The ICU Mobility Scale (IMS) is a tool with a good inter-rater reliability of 0.83 (95% CI: 0.76-0.90), has scores ranging from 0 to 10 according to the mobility activities, and allows scoring mobility of the patient from the moment in which he is bed-ridden until he independently walks without a walker and without the help of another person. In the IMS, higher scores are associated with greater mobility ^10^, this scale have been translated and validated to Portuguese with good reliability ^14^.

#### Translation and transcultural adaptation

This procedures were carried out between November and December 2016. In this phase, eight physiotherapists with experience in critical patients participated in addition to the certified translators. The certified translators (certified by the Ministry of Foreign Affairs of Colombia), were bilingual and native Spanish speakers, one of the translators (T1) had experience in translations in the medical field and the other translator (T2) had no translation experience in this field area. Each of them independently translated each scale into Spanish (T1 and T2). 

#### Translation synthesis (T1 + T2) "conciliation"

The translated versions were analyzed comparing item by item with the original scales in English by a physiotherapist with experience in critical patient care, bilingual and native Spanish speaker, arriving at a conciliated version.

This conciliated version was presented to a group of experts of 8 physiotherapists with a minimum experience of two years in critical care and intermediate English proficiency, who received the two formats of both scales translated into Spanish and the original format in English two weeks before the meeting for analysis and reflection. Questions and specific suggestions for change were sent to the main researcher and were analyzed and reviewed in a plenary, generating a corrected version in Spanish that was easy to understand for all participants. 

#### Re-translation

The corrected version of the scales in Spanish was translated (RT1 and RT2) back into English by two certified translators fluent in English and Spanish. These translators did not have contact with the original version of the scale in English nor did they know the initial translation process. The re-translation (Spanish to English) and the original English version of both scales were compared, and no significant differences were identified between them that would affect the meaning of the items. This re-translation was sent to the authors of each scale, to ensure that the Spanish version really reflected the content of the original version. 

#### Translation and cultural adaptation results

There was match between the translators during the drafting of the T1 and T2 versions. Based on the analysis of the notes issued at the expert meeting, recommendations arose related to practical aspects for the application of the two scales in Spanish (T1-T2):

- For the Perme Score.
- Could the graphic form of the domain items of the potential barriers in item 5 be modified? In practical terms, the statements are more visible if they are vertical.
- Could the instructions have more specific definitions to merge the assistance percentage during transfers?
- In the Gait item, could we convert distance units from feet to meters? In Colombia and a large part of Spanish-speaking countries, the unit of measurement commonly used for distance is meters.

Tables 1 and 2 describe the items in which discrepancies were identified among the translators, the suggestions of the physiotherapists in the T1-T2 version, and those made by the authors of the scales.

**Table 1:** Translation and final version of the Perme Score. Spanish version.

Original description	Translator 1	Translator 2	T1-2	Final Version
Perme Score Instructions	Perme ICU Mobility Score	Perme ICU Mobility Score	Instructions for the Perme ICU Mobility Score	Perme Score
2. "blink your eyes"	Blink	Blink	open and close your eyes	Open and close your eyes
5. "a drip"	Venocolysis	Venocolysis	Drips	Intravenous drips
7. "semi-recumbent position"	Semi-recumbent position	A partially reclined position.	Semi-lying position	Semi-lying position
9. "Supine to sit"	From supine to sitting	From supine to sitting	Supine to sitting	From supine to sitting
10. "Static sitting balance"	Static sitting balance	Static sitting balance	Static sitting balance	Static sitting balance
11. "Sit to stand"	From sitting to standing	From sitting to standing	Sitting to standing	From sitting to standing
12. "Static standing balance"	Static standing balance	Static standing balance once the position was established	Static standing balance	Static standing balance
15. "Endurance (distance walked in 2 minutes including sitting or standing rest periods"	Resistance (the distance walked in 2 minutes including the periods of sitting or standing rest	Resistance (the distance walked in 2 minutes including the periods of sitting or standing rest	Resistance (the distance walked in 2 minutes including the periods of sitting or standing rest	Resistance (the distance walked in 2 minutes including periods of sitting or standing rest

**Table 2:** Translation and final version of the ICU Mobility Scale (IMS)

Classification	Original description	Translator 1	Translator 2	Final version
3	Assistance	assistance	assistance	help
4	standing lifter device or title table	lifting device or tilt table	Lifting device or tilt table	stand-up device or stand-up table
5	standing lifter device	lifting device	Lifting device with wheels	standing device
6	marching on spot	static march	March in place	March in place
7-11	Yards	meters	meters	meters

#### Re-translation into english

The two versions of each scale retranslated to the English language were obtained. The divergences found between the original versions in English and the versions re-translated from Spanish were resolved by discussing with the research team, and the final version of both scales was obtained in Spanish. The original authors of both scales approved the final version in Spanish.

### Inter-rater reliability 

This process was carried out between February and June 2017. The final version of both scales was used to determine their inter-rater reliability. Initially 4 physiotherapists were trained in the application of both scales and a pilot test was carried out with a convenience sample with 30 patients. The approximate time for the application of the IMS was less than two minutes, and less than five minutes for the Perme Score.

The inter-rater reliability study was conducted in an adult 14-bed ICU in a hospital of the third level of complexity. The sample size was calculated using the advance estimate of Perme *et al* ^11^. The Kappa indices reported for this work ranged between 0.2748 and 0.9474 and the proportions of agreement were between 68.6% and 100% for the 15 items evaluated in this study. To calculate the sample size of the present study, the lowest proportion of reported agreement was taken (68.6%) and it was defined that the target Kappa index was 0.2784 or higher as than 0 to ensure that the calculated n was adequate. According to the above, the standard error for Kappa was calculated equal to zero with a confidence level of 95%, obtaining an EE: 0.1420. 

The sample size was calculated using the following formula:

$$n\;=\;\frac{0.6857}{\left(0.1420{)}^{2}\;(1-0.6857\right)}\;=\;108.2$$

Resulting in a sample size of 108 patients. Taking into account possible drop outs and that the selection process was performed by incomplete balanced blocks, a sample of 150 patients was considered convenient.

### Raters preparation and pilot study 

A 12-hour training was conducted for the 4 physiotherapists who were in charge of the data collection; each professional evaluated each patient and recorded the duration, execution, comments and questions generated during the process. Patients who were assessed in this pilot test had characteristics similar to those of the sample.

### Validation process 

To evaluate the inter-rater reliability of both scales, these were applied to 150 patients. Patients were over 18 years of age, with independent mobility before admission to the ICU (Barthel score above 90), reported by the closest relatives or by the patient (based on their health situation 7 days before admission to the ICU), we excluded patients with hearing loss, patients who were transferred from other units without clinical data, patients with re-admission to the ICU, patients with unstable fractures or injuries that prevented EM or who did not speak Spanish. All family members and patients, if the clinical condition allowed, signed informed consent prior to their participation in the study. The application of the scales was carried out at two different times (24 hours upon admission/discharge from the ICU).

Two pairs of trained raters were formed who independently applied the two scales at the same time for the same patient. Rater "A", specialist physiotherapist (over 5 years of experience) evaluated the patient, the rater "B", physiotherapist without specialization (with over 5 years of experience) observed the entire process. Both raters registered their results (in scoring forms) after completing the application of the two scales and before performing physiotherapy. 

The scoring forms of the two scales were completed separately, without communication between the raters. Rater "B" was responsible for recording the other variables of the study (sociodemographic, diagnosis, mechanical ventilation time, Apache II score (Acute Physiology And Chronic Health Evaluation II), time of stay in ICU, type of weaning, place of discharge). 

The two scales and the raters were randomized per incomplete balanced blocks using sealed envelopes. The main researcher and the ICU work team were masked. 

The institution's standardized EM checklist was used before the application of the scales, related to the indications, with hemodynamic, psychic and motor stability. 

### Inter-rater reliability process analysis

Once the information recorded in the collection formats was verified, it was entered into a database designed in Excel 2013. The SPSS 22 program was used for the statistical analysis.

For the description of the patients' clinical characteristics, the central trend, mean and median measures were used, the standard deviation (SD) was used as a measure of dispersion, and absolute frequencies and percentages (%) were used according to the type and distribution of each variable.

To assess the inter-rater reliability (observer/rater) for the scores of the IMS and Perme Score at both times, the intraclass correlation index (ICC) was used and 95% confidence intervals (95% CI) were determined with a level of significance of *p*: <0.05. The classification of Landis and Koch ^17^ was taken into account for the interpretation of the ICC values (0: Poor, 0.01-0.20: mild; 0.21-0.40: regular, 0.41-0.60: moderate; 0.61-0.80: Substantial, 0.81-1.00: Almost perfect). To verify the agreement between the raters, the Kappa index was used and interpreted as a direct relation from 0 to 1, where 0 is greater disagreement and 1 perfect agreement, qualitatively values greater than 0.8 were interpreted as excellent, greater than 0.7 strong, and greater than 0.6 good. Dispersion graphs were used between the measurements obtained by the observers and raters at each moment, and the Bland Altman graph was used in the end. The Kolmogorov test was used to test the normality of the distribution of the IMS and Perme Score.

## Results

### Patients' clinical characteristics

**Figure 1:**
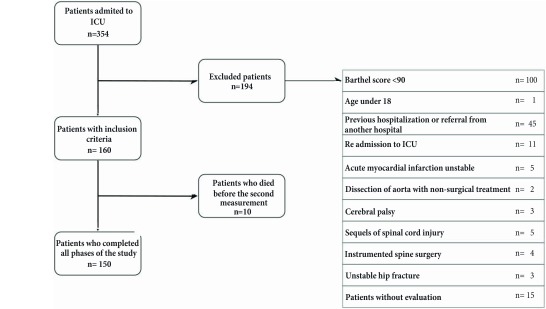
Recruitment and flow of participants.

During the data collection period, 150 patients were included. Figure 1 shows the recruitment and the flow of participants. For 5 months, 354 patients were admitted to the ICU; out of these, 194 presented exclusion criteria, 15 patients were not assessed. 

The clinical characterization is described in Table 3. Males were 52.0% and the average age was 58 ±17 years; 55.3% were hospitalized for clinical reasons, with cardiac issues being the most prevalent hospitalization reason. Invasive mechanical ventilation was present in 63 (42.0%) of the cases and easy weaning occurred in 81.0% of patients.

**Table 3:** Patients' clinical characteristics

Characteristics	Mean/Median/Percentages n = 150
Age (Mean/Range)	58.4 (18-92)
Male gender (%)	78 (52.0)
Reason for admission:	
Clinical	
Cardiac n (%)	40 (26.7)
Respiratory n (%)	14 (9.3)
Gastrointestinal n (%)	9 (6.0)
Neurological n (%)	1 (0.7)
Sepsis n (%)	7 (4.7)
Other n (%)	12 (8.0)
Surgical	
Cardiac n (%)	32 (21.3)
Neurosurgery n (%)	17 (25.4) 17 (11.3)
Trauma n (%)	4 (2.6)
Other n (%)	14 (9.3)
Invasive mechanical ventilation n (%)	63 (42.0)
Tracheostomy n (%)	8 (5.3)
Time (days) of mechanical ventilation Mean (95% CI)	2.8 (1.8-3.6)
Type of weaning	
Easy Weaning n (%)	51 (81.0)
Prolonged weaning n (%)	12 (19.0) *
Time (days) of weaning (Median, RIC) (Median, RIC)	1 (0-1)
Total time (days) in intensive care unit (Mean ± SD)	4.63 ± 5.93
Discharge	
Intermediate care unit (%)	127 (84.7)
Hospitalization (%)	9 (6.0)
Others (%)	14 (9.3)
Deceased (%)	10 (6.7)
Sedatives n (%)	32 (21.3)
Analgesics n (%)	32 (21.3)
Muscle relaxants n (%)	3 (2.0)
APACHE II (Mean ± SD)	15 ± 8 **

SD: Standard deviation;

* 5 Patients deceased;

** Measured in 97 patients,

IQR: Interquartile range.

In order to verify the inter-rater agreement (observer/rater), the intraclass correlation coefficient (ICC) was used with a confidence interval (CI) of 95%. Values close to one for the ICC were obtained in both scales and measurements in the two moments. This shows an excellent agreement between raters and observers for both scales.

Considering that the analysis in general terms could mask reliability problems between the scores of each observer/rater pair, it was necessary to analyze each pair at the two moments of the measurements. When analyzing the results of the IMS in pairs it was observed that an excellent ICC was obtained for all roles (observer/rater) for the first measurement, obtaining values of one (1) in three of the four rater-observer combinations, while only one of the couples obtained an intraclass correlation of 0.941, which corresponds to a high correlation. Results similar to those of the first measurement were found in the second measurement of the IMS. The intraclass correlation index was 1 in three of the four combinations of couples, and 0.96 in one of the pairs (Table 4).

**Table 4:** Intraclass Correlation Coefficient. ICU Mobility Scale (IMS). First and Second measurements in the two pairs of raters.

	Measurement 1 Admission to ICU		Measurement 2 Discharge from ICU	
Couple/role	ICC	95% confidence interval ICC	ICC	95% confidence interval ICC
Ft.1 - Rater Ft. The Observer	1.000	(1.000-1.000)	1.000	(1.000-1.000)
Ft. E1- Rater Ft. 1- Observer	0.941	(0.885-0.970)	1.000	(1.000-1.000)
Ft. E 2- Rater Ft. 2-Observer	1.000	(1.000-1.000)	1.000	(1.000-1.000)
Ft.2 - Rater Ft. E 2-Observer	1.000	(1.000-1.000)	0.959	(0.926-0.977)

For all ICC *p* <0.05

ICC: Intraclass Correlation Coefficient

Ft. E: Specialist physiotherapist with over 5 years of experience

Ft: Physiotherapist without specialization with over 5 years of experience

ICU: Intensive care unit

When analyzing the results of the Perme Score in pairs, it was found that an excellent ICC was obtained for the first measurement, with values of one (1) in two of the four rater-observer combinations, while two of the observer/rater combinations obtained an ICC of 0.999. 

Results similar to those of the first measurement were also found in the second measurement for the Perme Score. The ICC was excellent in three of the four combinations and 0.998 in one of the pairs (Table 5).

**Table 5:** Intraclass Correlation Coefficient. Perme Score First and Second Measurements in the two pairs of raters

	Measurement 1 Admission to ICU		Measurement 2 Discharge from the ICU	
Couple/role	ICC	IC 95% ICC	ICC	IC 95% ICC
Ft. 1 -Rater. FT. E. 1 Observer	0.999	(0.998-0.999)	1.000	(1.000-1.000)
Ft. E1- Rater. Ft. 1- Observer	0.999	(0.998-1.000)	0.998	(0.997-0.999)
Ft. E2-Rater. Ft.2-Observer	1.000	(1.000-1.000)	1.000	(1.000-1.000)
Ft.2-Rater Ft. E 2-Observer	1.000	(1.000-1.000)	1.000	(1.000-1.000)

For all ICC *p* <0.05

ICC: Intraclass Correlation Coefficient.

Ft. E: Specialist physiotherapist with over 5 years of experience

Ft: Physiotherapist without specialization with over 5 years of experience

ICU: Intensive care unit

Table 6 presents the concordance analysis for the IMS and the Perme Score between rater/observer upon admission to, and discharge from, the ICU. In both measurements the results of the Bland and Altman analysis showed a uniform behavior of the differences around zero. The Kappa Index presented values higher than 0.95 for both scales.

**Table 6:** Analysis of agreement between the ICU Mobility Scale (IMS) and Perme Score.

	Measurement 1 Admission to ICU			Measurement 2 Discharge from the ICU		
	Kappa Index	* Mean Differences	* 95% CI Mean Differences	Kappa Index	* Mean Differences	* 95% CI Mean Differences
ICU Mobility Scale (IMS)	0.988	-0.007	-0.153, 0.167	0.992	-0.035	-0.858, 0.787
Perme Score	0.967	-0.013	-0.333, 0.307	0.985	0.028	- 0.491, 0.542

* Bland and Altman analysis results

## Discussion

The present work represents the first report of translation, cultural adaptation and inter-rater reliability of the Perme Score and the IMS in Spanish. The authors consider that since currently there are not valid scales in Spanish to assess functional mobility in the ICU context, the results of this research may be appropriate for teams of Spanish-speaking health care professionals and institutions to guide early mobilization interventions in the critical patient. 

The recommendations of a protocol were followed in the Spanish translation and the cultural adaptation of the Perme Score and the IMS, and both scales are easy to apply in critical patients. Translations and cultural adaptations of both scales exist for several countries, which facilitates the comparison between the studies carried out ^14^ ^,^ ^15^. 

The sample size (n = 150) in this study is greater than that used in the validation of the original versions of both scales, Nawa *et al*.^18^, (n= 20) and Hodgson et al. ^19^, (n= 100); and than the translation and validation to Portuguese (n= 103). 

The age, sex and medical diagnoses of the present study's sample are very similar to the demographic variables of the samples used in the validation studies ^14^ ^,^ ^18^ ^,^ ^19^. But unlike the other studies, information on the level of functionality prior to admission to the ICU was inquired through the Barthel Index, because the authors consider that this avoided selection biases that could interfere with the results.

The sample was recruited in patients with different medical diagnoses and the severity was classified upon admission to the ICU with the APACHE II. 

The original validation studies of the Perme Score and the IMS also report the use of APACHE II. Hodgson *et al* **.** ^19^, an average APACHE II score of 19 (SD 7) found; Nawa *et al*. ^18^, a median of 16.5 report with an Interquartile Range (IQR) of 7 to 30 points. In our study, we found an average score of 15 (SD 8); results similar to those reported in the original studies, suggesting that in the three studies the scales were used in patients in a wide range of severity

Parry *et al.* ^6^, mention that the relevant measurement properties to consider when selecting an instrument include, among others, the ability to obtain accurate results within or between the raters (intra- and inter-rater reliability, respectively).

In the reviewed publications, no studies were identified that assessed the inter-rater reliability in two pairs in the rater/observer role, in two different moments. Hodgson *et al*. ^19^, evaluated the inter-rater reliability of the IMS, between a nurse, an expert physiotherapist and a junior physiotherapist, evaluating each patient within 30 min of each other on the same day. Nawa *et al*. ^18^, evaluated the inter-rater reliability of the Perme Score, but in a single moment and with a single pair of raters (rater "A" evaluated the patient and rater "B" observed the entire process). Kawaguchi et al. also evaluated the inter-rater reliability of both scales following the same methodology reported by Nawa *et al* ^14^ ^,^ ^18^ ^,^ ^19^. High reliability values were reported in all the previous studies.

To identify the inter-rater reliability, the measurements were made by two pairs of raters made up of a physiotherapist without specialization (Ft.) with over 5 years of experience and a physiotherapist specializing in cardiopulmonary physiotherapy (Ft.E) with over 5 years of experience; each pair at two different times (24 hours upon admission to/discharge from the ICU), with a randomization of the order of the observer/rater role in each pair, made the measurements without knowing the scores obtained.

These methods differ from the validation studies of the original scales; the authors consider that changing roles during assessments favored reducing biases related to commands, approach and orientations to patients, which are factors that may be different from one professional to another and influence the scores. They also assume that a greater number of raters who rotate the rater/observer role represent a situation closer to what happens in an ICU with different professionals assessing the patient at different times, which makes our results applicable.

In this study we observed a high concordance and absence of significant differences between the observer/rater roles for the two scales in the two measurement moments. The authors of the research consider that this could be related to the training carried out, the appropriate learning and the clinical experience.

For the authors, it is important to note that, unlike the original validation processes of both scales, making the measurements at two different times allowed discriminating between the environments of ICU admission and the successive changes in the mobility of patients in the ICU context. They also consider that the randomization in the observer/rater role avoided the bias that the assessment could be conditioned by the recall effect of the first.

The study's methodological strengths are related to the randomization per incomplete balanced blocks to establish the rater/observer role, achieving a similar number of patients in each pair; with the heterogeneity in the patients' severity; with the strategies implemented to reduce the possibility of information and selection bias, and to control confusion factors, such as the masking of the main researcher and the measurements results and the quality control carried out to the information collection and analysis processes. 

The study's authors identified the following limitations: having been carried out in a single institution, not having taken into account other professionals of the ICU team (who could use the scales) and the absence of other functional assessments such as muscle strength. However, these aspects do not affect the validity of the results, bearing in mind that this study did not evaluate the effects of EM interventions. 

Further research is recommended regarding the use of scales to evaluate early mobility protocols, and to analyze other clinimetric properties of both scales. Although the translation was done in Colombia, it is feasible to use it in other Spanish-speaking countries, since the translation and cultural adaptation process took into account that the final Spanish version was the reflection of the original version in English, and that it was easy to understand, avoiding the use of ambiguous terms or local idioms ^20^ ^,^ ^21^.

## Conclusions

The process of translation, cultural adaptation and reliability of the Perme Score and the IMS was carried out with a rigorous methodology, and both scales are available in Spanish with an excellent inter-rater reliability at two different times (24 hours upon admission/discharge from the ICU). The use of both tools is recommended in the critical patient care context to guide and quantify EM interventions. 
