# Supplementary material for: Perme ICU Mobility Score (Perme Score) and the ICU Mobility Scale (IMS): translation and cultural adaptation for the Spanish language
Source: Colomb Med (Cali). 2018 Dec 30;49(4):265–72. doi: 10.25100/cm.v49i3.4042 (PMC6342087; doi:10.25100/cm.v49i3.4042)
Supplement: Related texts

# Original article: Escalas Perme ICU Mobility Score (Perme Score) e ICU Mobility Scale (IMS): traducción y adaptación cultural para el idioma español

## Introducción

Las actividades de movilización temprana en las unidades de cuidado intensivo (UCI) son una estrategia para mejorar la recuperación funcional durante y después de la enfermedad critica prolongada, disminuir la debilidad muscular adquirida en cuidado intensivo (DMA UCI), el delirio y también para disminuir la estancia hospitalaria ^1^^-^^3^

Las medidas de resultado descritas en las investigaciones sobre la movilidad en el paciente crítico han sido la duración de días en UCI y en el hospital, la duración de la ventilación mecánica, la fuerza muscular, la funcionalidad y la mortalidad ^4^^,^^5^. Sin embargo, ninguno de estos resultados constituye evaluaciones específicas y sensibles de la situación de movilidad de los pacientes. 

Un total de 26 instrumentos para medir la función de los pacientes críticos fue identificado en una revisión sistemática ^6^; sin embargo, solo seis instrumentos fueron diseñados específicamente para medir el estado funcional en pacientes críticos con un concepto relacionado con la movilidad, tales como: Physical Function in Intensive Care Test scored (PFIT-s), Functional *Status Score for the ICU* (FSS-ICU), Chelsea Critical Care Physical Assessment Tool (CPAx), ICU Mobility Scale (IMS), Perme *Intensive Care Unit Mobility Score (Perme Score),* Surgical Intensive Care Unit Optimal Mobility Scale (SOMS), y fueron originalmente construidos en inglés ^7^^-^^13^.

Tanto la IMS como el *Perme Score* han demostrado niveles adecuados de confiabilidad al aplicarse en diversas poblaciones de pacientes hospitalizados en UCI, en los últimos dos años se han llevado a cabo estudios de traducción y adaptación cultural al portugués y al alemán ^14^^,^^15^.

Ante la creciente utilización de la movilización temprana (MT) de pacientes en la UCI ^9^^,^^10^^,^^13^, existe una necesidad en países de habla hispana de tener instrumentos culturalmente válidos para medir de manera longitudinal la función física del paciente en la UCI. Hasta la fecha no se conocen en nuestra región, estudios que describan el estado de la movilidad funcional de los pacientes al egreso de la UCI, probablemente por no disponer de herramientas confiables y válidas para evaluar el estado de movilidad durante la enfermedad crítica.

 El uso de un instrumento que ya existe en un idioma y cultura de otro país, antes de ser implementado, debe pasar por un proceso de adaptación cultural y validación para verificar si la nueva versión es reproducible y confiable ^6^.

Disponer de instrumentos para medir la movilidad permitirá mejorar la identificación temprana de deficiencias, el inicio de la intervención y la provisión de servicios que reduzcan el impacto de la hospitalización en la independencia funcional y 

calidad de vida del paciente. El objetivo del estudio fue realizar la traducción, adaptación cultural y medir confiabilidad inter evaluador de la versión en español del Perme Score y de la IMS.

## Materiales y Métodos

### Pacientes y Métodos

Estudio de traducción y validación siguiendo los criterios de la lista de chequeo COSMIN (Consensus-based Standards for the Selection of Health Measurement Instruments) ^16^. Los autores de la versión original de las dos escalas dieron autorización para el proceso. Este estudio tuvo la aprobación del Comité de Ética Humana del Universidad del Valle y del Comité de la Institución (002-005) donde se realizó la investigación en el periodo de noviembre de 2016 y julio de 2017

### Descripción de las escalas

El “Perme Intensive Care Unit Mobility Score” (Perme Score), tiene buena confiabilidad entre evaluadores (ICC 0.98801) ^13^, y fue traducido y validado al portugués en pacientes de UCI (confiabilidad inter evaluador (κ: >0.9 e: >0.9 para la mayor parte de los dominios). Contiene 15 ítems agrupados en 7 categorías: estado mental, barreras potenciales para la movilidad, fuerza funcional, movilidad en cama, transferencias, marcha y resistencia. La puntuación varía de 0 a 32; utiliza un rango máximo de 2 a 4 puntos para cada uno de los 15 ítems incluidos. Un puntaje total alto indica pocas barreras para la movilidad y que se requiere mínima asistencia para actividades de movilidad ^11^.

La ICU Mobility Scale (IMS), es una herramienta con buena confiabilidad inter evaluador de 0.83 (IC 95%: 0.76-0.90), tiene puntaciones que varían entre 0 y 10 de acuerdo a actividades de movilidad, y permite calificar la movilidad del paciente desde que está en cama hasta que de manera independiente camina sin caminador y sin ayuda de otra persona. En la IMS las puntuaciones mayores están asociadas a mayor movilidad ^10^ y ha sido traducida y validada al portugués con buena confiabilidad ^14^. 

#### Traducción y adaptación transcultural

Fue realizada en noviembre y diciembre del 2016. En esta fase participaron además de los traductores oficiales, ocho fisioterapeutas con experiencia en pacientes críticos.

Los traductores oficiales (certificados por el Ministerio De Relaciones Exteriores De Colombia), eran bilingües y hablantes nativos de español, uno de los traductores (T1) tenía experiencia en traducciones en el área de salud y el traductor (T2) sin experiencia en traducciones en el área. Cada uno de ellos tradujo de manera independiente cada escala al idioma español (T1 y T2). 

#### Síntesis de la traducción (T1 + T2) “conciliación”

Las versiones traducidas fueron analizadas comparando ítem por ítem con las escalas originales en inglés por un fisioterapeuta con experiencia en atención de pacientes críticos, bilingüe y hablante nativo de español, llegando a una versión conciliada.

Esta versión conciliada fue presentada a un grupo de expertos de 8 fisioterapeutas con experiencia mínima de 2 años en atención de pacientes críticos y proficiencia intermedia en inglés, quienes dos semanas antes de realizarse la reunión, recibieron los 2 formatos de ambas escalas traducidas al español y el formato original en inglés para su análisis y reflexión. Las dudas y las propuestas específicas se enviaban al investigador principal y en plenaria se analizaron y revisaron generándose una versión corregida en idioma español que fuera de fácil comprensión. 

#### Re traducción

La versión corregida de las escalas en español fue traducida (RT1 y RT2) al inglés por dos traductores certificados con fluidez en los idiomas inglés y español. Estos traductores no tuvieron contacto con la versión original de la escala en inglés ni conocieron el proceso inicial de traducción. La retraducción (español a inglés) y la versión original en inglés de ambas escalas fueron comparadas y no se identificaron diferencias importantes entre ellas que afectaran el significado de los ítems. Esta retradución fue enviada a los autores de cada 

escala, para asegurar que la versión en español reflejara realmente el contenido de la versión original. 

#### Resultados de Traducción y adaptación cultural 

Hubo concordancia entre los traductores durante la preparación de las versiones T1 y T2. A partir del análisis de las notas emitidas en la reunión de expertos surgieron recomendaciones relacionadas con aspectos prácticos para la aplicación de las dos escalas en idioma español (T1-T2):

Para el Perme Score.

- ¿Se podría modificar la forma gráfica de los ítems del dominio de las potenciales barreras ítem 5? En términos prácticos se hacen más visible los enunciados en forma vertical.
- Se podría tener en el instructivo definiciones más específicas para unificar el porcentaje de asistencia durante las trasferencias.?
- ¿Se podrían cambiar en el ítem de Marcha, en la distancia los pies por metros? En Colombia y gran parte de países de habla hispana la unidad de medida empleada habitualmente para la distancia es metros.

En las Tablas 1 y 2 se describen los ítems en los cuales fueron identificadas discrepancias entre los traductores, las sugerencias de los fisioterapeutas en la versión T1-T2 y las realizadas por los autores de las escalas.

**Tabla 1:** Traducción por traductores y versión final del Perme Score. Versión en español.

Descripción original	Traductor 1	Traductor 2	T1-2	Versión definitiva
Perme Score Instructions	Puntaje de movilidad en la UCI de Perme	Puntaje de movilidad en la UCI de Perme	Instrucciones del Puntaje de movilidad en unidades de cuidados intensivos de Perme	Perme Score
2. "blink your eyes"	Parpadear	parpadear	abra y cierra los ojos	Abra y cierra los ojos
5. "a drip"	Venoclisis	Venoclisis	Goteos	Goteos intravenosos
7. "semi-recumbent position"	Posición semi decúbito	Una posición parcialmente reclinada.	Posición semi acostado	Posición semi-acostado
9."Supine to sit"	De supino a sedente	De supino a sedente	Supino a sedente	De supino a sentado
10."Static sitting balance"	Equilibrio en sedente estático	Equilibrio en sedente estático	Equilibrio estático en sedente	Equilibrio estático sentado
11."Sit to stand"	De sedente a bípedo	De sedente a bípedo	Sedente a bípedo	De sentado a parado
12."Static stading balance"	Equilibrio en bípedo estático	Equilibrio en bípedo estático una vez se estableció la posición	Equilibrio estático en bípedo	Equilibrio estático parado
15."Endurance (distance walked in 2 minutes including sitting or standing rest periods"	La resistencia (la distancia caminada en 2 minutos incluyendo los periodos de descanso en sedente o bípedo	La resistencia (la distancia caminada en 2 minutos incluyendo los periodos de descanso en sedente o bípedo	La resistencia (la distancia caminada en 2 minutos incluyendo los periodos de descanso en sedente o bípedo	La resistencia (la distancia caminada en 2 minutos incluyendo los periodos de descanso sentado o parado

**Tabla.2. Traducción:** por traductores y versión final de la ICU Mobility Scale (IMS)

Clasificación	Descripción original	Traductor 1	Traductor 2	Versión definitiva
3	Assistance	asistencia	asistencia	ayuda
4	standing lifter device or title table	dispositivo elevador o mesa basculante	aparato levantador o una mesa basculante	dispositivo bipedestador o mesa de bipedestación
5	standing lifter device	dispositivo de elevación	Dispositivo elevador con ruedas	dispositivo bipedestador
6	marching on spot	marcha estática	Marchar en el lugar	Marcha en el puesto
7-11	Yardas	metros	metros	metros

#### Re traducción al idioma inglés

Se obtuvieron las dos versiones de cada escala retraducidas al idioma inglés. Las divergencias encontradas entre las versiones originales en idioma inglés y las versiones retraducidas desde el idioma español se resolvieron mediante discusión del equipo investigador, y se obtuvo la versión final de ambas escalas en idioma español. Los autores originales de ambas escalas aprobaron la versión final en idioma español.

### Confiabilidad Inter-evaluador 

Este proceso fue realizado entre febrero y junio de 2017. La versión final de ambas escalas, fue utilizada para determinar la confiabilidad inter-evaluador. Inicialmente 4 fisioterapeutas fueron entrenados en la aplicación de ambas escalas y se realizó una prueba piloto con una muestra por conveniencia con 30 pacientes. El tiempo aproximado para la aplicación de la IMS fue menor de dos minutos y menos de cinco minutos para el Perme Score.

El estudio de confiabilidad interevaluador se realizó en una UCI adulto de 14 camas en una clínica de tercer nivel de complejidad. El tamaño muestral se calculó utilizando la estimación adelantada de Perme et al,^11^. En este trabajo se reportaron para los 15 ítems evaluados los índices de Kappa que oscilaron entre 0.2748 y 0.9474 y las proporciones de concordancia entre 68.6% y 100%. Para el cálculo de tamaño de muestra del presente estudio se tomó la menor proporción de concordancia reportada (68.6%) y se definió que se deseaba identificar como diferente de 0 un índice Kappa 0.2784 o superior para garantizar que el n calculado fuera adecuado. De acuerdo a lo anterior se calculó el error estándar para Kappa igual a cero con un nivel de confianza del 95%, obteniendo un EE= 0.1420. 

El tamaño de la muestra se calculó utilizando la siguiente fórmula:

$$n\;=\;\frac{0.6857}{\left(0.1420{)}^{2}\;(1-0.6857\right)}\;=\;108.2$$

Dando como resultado un tamaño de muestra de 108 pacientes. Teniendo en cuenta las posibles pérdidas y que el proceso de selección fue realizado por bloques incompletos balanceados, se consideró conveniente una muestra de 150 pacientes.

### Preparación de evaluadores y estudio Piloto 

Se realizó un entrenamiento de 12 horas, para los 4 fisioterapeutas que estuvieron a cargo de la recolección de datos; cada profesional valoró a cada paciente y se registró la duración, ejecución, comentarios y preguntas generadas durante el proceso. Los pacientes que se valoraron en esta prueba piloto tuvieron características similares a los de la muestra.

### Proceso de validación 

Para evaluar la confiabilidad inter evaluador de ambas escalas, éstas se aplicaron a 150 pacientes. Fueron incluidos pacientes con edad mayor de 18 años, con movilidad independiente antes de ingreso a UCI (Puntuación de Barthel por encima de 90), reportado por los familiares más próximos o por el propio paciente en función de la situación de salud del paciente 7 días al ingreso a UCI), y fueron excluidos pacientes con deficiencia auditiva, pacientes con transferencia de otras unidades sin datos clínicos, pacientes con re internación a UCI, pacientes con fracturas inestables o lesiones que impidieran la MT o que no hablaran español.Todos los familiares y los pacientes, si la condición clínica lo permitía, firmaron consentimiento informado previo a su participación en el estudio. La aplicación de las escalas se realizó en dos momentos diferentes (24 horas al ingreso/egreso de UCI).

Se conformaron dos parejas de evaluadores entrenados quienes independientemente aplicaron las dos escalas al mismo tiempo para el mismo paciente. El evaluador "A”, fisioterapeuta especialista (mayor de 5 años de experiencia) evaluaba el paciente, el evaluador "B", fisioterapeuta sin especialización (con más de 5 años de experiencia) observaba todo el proceso. Ambos evaluadores completaron el formato de recolección de la **información** después de terminar la aplicación de las dos escalas y antes de realizar la fisioterapia. 

Los formatos de puntuación de las dos escalas fueron diligenciados separadamente, sin comunicación entre los evaluadores. El evaluador "B" fue el responsable de registrar las otras variables del estudio (sociodemográficas, diagnóstico, tiempo de ventilación mecánica, puntuación de Apache II (Acute Physiology And Chronic Health Evaluation II), tiempo de estancia en UCI, tipo de destete, lugar de egreso). 

Las dos escalas y los evaluadores fueron aleatorizados por bloques incompletos balanceados utilizando sobres cerrados. Se realizó enmascaramiento del investigador principal y del equipo de trabajo de UCI. 

Antes de la aplicación de las escalas se utilizó la lista de chequeo de MT estandarizada en la institución, relacionada con las indicaciones, con estabilidad hemodinámica, psíquica y motora. 

### Análisis del proceso de confiabilidad inter evaluador

Una vez verificada la información consignada en los formatos de recolección, se digitó en una base de datos diseñada en el programa Excel versión 2013. Para el análisis estadístico fue utilizado el programa SPSS 22.

Para la descripción de las características clínicas de los pacientes se utilizaron las medidas de tendencia central, media y mediana, como medida de dispersión la desviación estándar (DE), y se utilizaron frecuencias absolutas y porcentajes (%) de acuerdo al tipo y distribución de cada variable.

Para evaluar la confiabilidad ínter evaluador (observador/evaluador) para las puntuaciones de la IMS y Perme Score en los dos momentos, se utilizó el índice de correlación intraclase (ICC) y se determinaron intervalos de confianza del 95% (IC 95%), con un nivel de significancia de *p* <0.05. Para la interpretación de los valores del ICC se tuvo en cuenta la clasificación de Landis y Koch ^17^ (0: Pobre, 0.01-0.20: leve; 0.21-0.40: regular, 0.41-0.60: moderado; 0.61-0.80: Substancial, 0.81-1.00: Casi perfecto). Para verificar la concordancia entre los evaluadores se utilizó el índice de Kappa y se interpretó como una relación directa de 0 a 1, donde 0 es mayor desacuerdo y 1 concordancia perfecta, cualitativamente valores mayores de 0.8 fueron interpretados como excelente, mayores de 0.7 fuerte, y mayores de 0.6 bueno. Se usaron gráficos de dispersión entre las mediciones obtenidas por los observadores y evaluadores en cada momento, finalmente se usó el gráfico de Bland Altman. Para probar la normalidad de la distribución de la IMS y Perme Score se usó la prueba de Kolmogorov.

## Resultados

### Características clínicas de los pacientes

Durante el periodo de recolección de datos, se incluyeron 150 pacientes. La Figura 1 muestra el reclutamiento, y el ﬂujo de participantes. Durante 5 meses 354 pacientes ingresaron a UCI; de estos, 194 presentaron criterios de exclusión, 15 pacientes no fueron evaluados. 

**Figura 1:** Reclutamiento y flujo de participantes.

La caracterización clínica se describe en la Tabla 3. El 52.0% correspondió al género masculino y la edad promedio fue de 58 ±17 años. El 55.3% estaban internados por aspectos clínicos siendo el cardiaco el motivo de internación más prevalente. La ventilación mecánica invasiva estuvo presente en 63 (42.0%) de los casos y el destete fácil ocurrió en 81.0% de los pacientes.

**Tabla 3:** Características clínicas de los pacientes

Características	Media/ Mediana/Porcentajes n=150
Edad (Media /Rango)	58.4 (18-92)
Sexo masculino (%)	78 (52.0)
Motivo da admisión:	
**Clínico**	
Cardíaco n(%)	40 (26.7)
Respiratorio n (%)	14 (9.3)
Gastrointestinal n (%)	9 (6.0)
Neurológico n (%)	1 (0.7)
Sepsis n (%)	7 (4.7)
Otros n (%)	12(8.0)
**Quirúrgico**	
Cardíaco n (%)	32 (21.3)
Neuroquirúrgico n (%)	17 (25.4) 17 (11.3)
Trauma n (%)	4 (2.6)
Otros n (%)	14 (9.3)
Ventilacion mecánica invasiva n (%)	63 (42.0)
Traqueostomía n (%)	8 (5.3)
Tiempo (días) de ventilación mecánica Media (IC 95%)	2.8 (1.8-3.6)
Tipo de destete	
Destete Fácil n (%)	51 (81.0)
Destete Prolongado n (%)	12 (19.0) *
Tiempo (días) de destete (Mediana, RIC) (Mediana, RIC)	1 (0-1)
Tiempo (días) total en unidad de cuidado intensivo (Media ± DE)	4.63 ± 5.93
Egreso	
Unidad de cuidado intermedio (%)	127 (84.7)
Hospitalización (%)	9 (6.0)
Otros (%)	14 (9.3)
Fallecidos (%)	10 (6.7)
Sedantes n (%)	32 (21.3)
Analgésicos n (%)	32 (21.3)
Relajantes musculares n (%)	3 (2.0)
APACHE II (Media ± DE)	15 ± 8**

DE: Desviación Estándar;

*5 Pacientes fallecidos;

** Se midió en 97 pacientes,

RIC: Rango Intercuartílico.

Para verificar la concordancia interevaluador (observador/evaluador) se usó el coeficiente de correlación intraclase (ICC) con un intervalo de confianza (IC) del 95%. En ambas escalas y mediciones en los dos momentos se obtuvieron valores cercanos a uno para el ICC. Lo que muestra una excelente concordancia entre evaluadores y observadores para ambas escalas.

Considerando que el análisis en términos generales podía enmascarar problemas de confiabilidad entre las puntuaciones realizadas por cada pareja observador/ evaluador, fue necesario realizar el análisis de cada pareja en los dos momentos de las mediciones.

Al analizar los resultados de la IMS por parejas se observó que para la primera medición se obtuvo un ICC excelente para todos roles (observador/evaluador), obteniéndose valores de uno (1) en tres de las cuatro combinaciones evaluador-observador, mientras que sólo una de las parejas obtuvo una correlación intraclase de 0.941, lo que corresponde a una alta correlación. Para la segunda medición de la IMS se encontraron resultados similares a los de la primera medición. El índice de correlación intraclase fue de 1 en tres de las cuatro combinaciones de parejas, y de 0,96 en una de las parejas (Tabla 4).

**Tabla 4:** Coeficiente de Correlación Intraclase. ICU Mobility Scale (IMS). Primera y Segunda medición en las dos parejas de evaluadores.

	Medición 1 Ingreso a UCI		Medición 2 Egreso de UCI	
Pareja/rol	ICC	Intervalo de confianza del 95% ICC	ICC	Intervalo de confianza del 95% ICC
Ft.1 -Evaluador. Ft. E1 Observador	1.000	(1.000-1.000)	1.000	(1.000-1.000)
Ft. E1- Evaluador. Ft. 1- Observador	0.941	(0.885-0.970)	1.000	(1.000-1.000)
Ft. E 2-Evaluador. Ft. 2-Observador	1.000	(1.000-1.000)	1.000	(1.000-1.000)
Ft.2 -Evaluador. Ft. E 2-Observador	1.000	(1.000-1.000)	0.959	(0.926-0.977)

Para todos los ICC *p* <0.05

ICC: Coeficiente de Correlación Intraclase

Ft. E: Fisioterapeuta especialista con más de 5 años de experiencia

Ft: Fisioterapeuta sin especialización con más de 5 años de experiencia

UCI: Unidad de cuidado intensivo

Al analizar los resultados del Perme Score por parejas se observó que para la primera medición se obtuvo un ICC excelente, obteniendo valores de uno (1) en dos de las cuatro combinaciones evaluador-observador, mientras que dos de las combinaciones observador/evaluador obtuvieron un ICC de 0.999. 

En el caso de la segunda medición para el Perme Score también se encontraron resultados similares a los de la primera medición. El ICC fue excelente en tres de las cuatro combinaciones y de 0.998 en una de las parejas (Tabla 5).

**Tabla 5:** Coeficiente de Correlación Intraclase. Perme Score. Primera y Segunda Medición en las dos parejas de evaluadores

	Medición 1 Ingreso a UCI		Medición 2 Egreso a UCI	
Pareja/rol	ICC	IC 95% ICC	ICC	IC 95% ICC
Ft. 1 -Evaluador. FT. E. 1 Observador	0.999	(0.998-0.999)	1.000	(1.000-1.000)
Ft. E1- Evaluador. Ft. 1- Observador	0.999	(0.998-1.000)	0.998	(0.997-0.999)
Ft. E2-Evaluador. Ft.2-Observador	1.000	(1.000-1.000)	1.000	(1.000-1.000)
Ft.2-Evaluador. Ft. E 2-Observador	1.000	(1.000-1.000)	1.000	(1.000-1.000)

Para todos los ICC *p*: <0.05

ICC: Coeficiente de Correlación Intraclase.

Ft. E: Fisioterapeuta especialista con más de 5 años de experiencia

Ft: Fisioterapeuta sin especialización con más de 5 años de experiencia

UCI: Unidad de cuidado intensivo

En la Tabla 6, se presenta el análisis de concordancia para el IMS y el Perme Score entre evaluador/observador al ingreso y egreso de UCI. En las dos mediciones los resultados del análisis Bland y Altman mostraron un comportamiento uniforme de las diferencias alrededor de cero. El Índice de Kappa presentó valores mayores de 0.95 para ambas escalas.

**Tabla 6:** Análisis de concordancia entre la ICU Mobility Scale (IMS) y Perme Score.

	Medición1 Ingreso a UCI			Medición 2 Egreso a UCI		
	Índice Kappa	*Media Diferencias	*IC 95% Media Diferencias	Índice Kappa	*Media Diferencias	*IC 95% Media Diferencias
ICU Mobility Scale (IMS)	0.988	-0.007	-0.153, 0.167	0.992	-0.035	-0.858, 0.787
Perme Score	0.967	-0.013	-0.333, 0.307	0.985	0.028	- 0.491, 0.542

*Resultados análisis Bland y Altman

## Discusión

El presente trabajo representa el primer reporte de traducción, adaptación cultural y confiabilidad inter evaluador del Perme Score y de la IMS en idioma español, los autores consideran que teniendo en cuenta que a la fecha, en nuestro medio no hay escalas traducidas ni validadas para su uso en español que permitan evaluar la movilidad funcional en el contexto de la UCI, los resultados de esta investigación pueden ser apropiados por los equipos de profesionales e instituciones de lengua hispana para guiar intervenciones de movilización temprana en el paciente crítico. 

En la traducción para el idioma español y la adaptación cultural del Perme Score y de la IMS se siguieron las recomendaciones de un protocolo, ambas escalas son de fácil aplicación en pacientes críticos. Existen traducciones y adaptaciones culturales de ambas escalas para varios países lo que facilita la comparación entre los estudios realizados ^14^^,^^15^. 

El tamaño de muestra (n= 150) en este estudio es mayor al utilizado en la validación de las versiones origínales de ambas escalas, Nawa *et al*.^18^, (n= 20) y Hodgson *et al*.^19^, (n= 100); y al proceso de traducción y validación al idioma portugués (n= 103). 

La edad, el sexo y los diagnósticos médicos de la muestra del presente estudio son muy similares a las variables demográficas de las muestras usadas en los estudios de validación ^14^^,^ ^18^^,^^19^. Pero a diferencia de los otros estudios, en el presente se indagó atraves del Índice de Barthel información del nivel de funcionalidad previo al ingreso a UCI, los autores consideran que lo anterior evitó sesgos de selección que pudieran interferir con los resultados.

La muestra se reclutó en pacientes con diferentes diagnósticos médicos y se clasificó la severidad o gravedad al ingreso a UCI con el APACHE II. 

Los estudios originales de validación del Perme Score y de la IMS también reportan el uso del APACHE II. Hodgson *et al* **.**^19^, encontraron un puntaje promedio del APACHE II de 19 (DE 7); Nawa *et al*. ^18^, reportan mediana de 16.5 con un Rango Intercuartílico (IQR) de 7 a 30 puntos. En nuestro estudio se encontró puntaje promedio de 15 (DE 8); resultados similares a los reportados en los estudios originales, lo que sugiere que en los tres estudios se utilizaron las escalas en pacientes en un rango amplio de gravedad

Parry *et al*. ^6^, mencionan que las propiedades de medición relevantes a considerar al seleccionar un instrumento incluyen entre otras, la capacidad de obtener resultados precisos dentro o entre los evaluadores (confiabilidad intra e inter-evaluadores, respectivamente).

En las publicaciones revisadas no se identificaron estudios que evaluaran la confiabilidad interevaluador en dos parejas en el rol de evaluador/observador, en dos momentos diferentes. Hodgson *et al*. ^19^, evaluaron la confiabilidad inter evaluador de la IMS, entre una enfermera, un fisioterapeuta experto y un fisioterapeuta junior, evaluando cada paciente dentro de 30 min el uno del otro en el mismo día. Nawa et al. ^18^, evaluaron la confiabilidad inter evaluador del Perme Score, pero en un solo momento y con una sola pareja de evaluadores (evaluador "A" evaluaba al paciente y evaluador "B" observaba todo el proceso). Kawaguchi y cols también evaluaron la confiabilidad inter evaluador de ambas escalas siguiendo la misma metodología reportada por Nawa *et al*^14^^,^^18^^,^^19^. En todos los anteriores estudios fueron reportados valores altos de confiabilidad.

Para identificar la confiabilidad inter evaluador, las mediciones fueron realizadas por dos parejas de evaluadores conformadas por un fisioterapeuta sin especialización (Ft.) con más de 5 años de experiencia y por un fisioterapeuta especialista en fisioterapia cardiopulmonar (Ft.E) con más de 5 años de experiencia; cada pareja en dos momentos diferentes (24 horas al ingreso/egreso de UCI), con una aleatorización del orden del rol observador/evaluador en cada pareja, realizaron las mediciones desconociendo la puntuaciones obtenidas.

Estos métodos difieren con los estudios de validación de las escalas originales; los autores consideran que cambiar de rol durante las evaluaciones favoreció a disminuir los sesgos relacionados con los comandos, abordaje y orientaciones a los pacientes, que son factores que pueden ser diferentes de un profesional a otro e influir en las puntuaciones. También asumen que un mayor número de evaluadores que rotan el rol evaluador/observador representa una situación más cercana a lo que ocurre en una UCI con diferentes profesionales valorando al paciente en diferentes momentos, lo que hace que nuestros resultados sean aplicables.

En este estudio se observó una alta concordancia y ausencia de diferencias significativas entre los roles observador/ evaluador para las dos escalas en los dos momentos de medición. Los autores de la investigación consideran que lo anterior, pudo estar relacionado con el entrenamiento realizado, el aprendizaje adecuado y la experiencia clínica.

Para los autores, es importante resaltar que, a diferencia de los procesos originales de validación de ambas escalas, realizar las mediciones en dos momentos diferentes, permitió discriminar entre los entornos de ingreso a UCI y los cambios sucesivos en la movilidad de los pacientes en el contexto de la UCI. También consideran que la aleatorización en el rol observador/ evaluador evitó el sesgo de que la evaluación pudiese estar condicionada por el efecto del recuerdo de la primera.

Las fortalezas metodológicas del estudio están relacionadas con la aleatorización por bloques balanceados incompletos para establecer el rol de evaluador/observador lográndose garantizar un número similar de pacientes en cada pareja; con la heterogeneidad en la gravedad de los pacientes; con las estrategias implementadas para disminuir la posibilidad de sesgos de información y selección, y para controlar factores de confusión, tales como el enmascaramiento del investigador principal y de los resultados de las mediciones y el control de calidad realizado a los procesos de recolección y análisis de la información. 

Los autores del estudio consideran que entre las limitaciones se identifican: el haberse realizado en una sola institución, no haber tenido en cuenta otros profesionales del equipo de la UCI (quienes podrían llegar a utilizar las escalas) y la ausencia de otras evaluaciones funcionales como fuerza muscular. Sin embargo, estos aspectos no afectan la validez de los resultados, teniendo en cuenta que este estudio no evalúo los efectos de las intervenciones de MT. 

Se recomienda la realización de futuras investigaciones con el uso de las escalas para evaluar la aplicación y validación de protocolos de movilidad temprana, también se reconoce la necesidad de analizar otras propiedades clinimétricas. Aunque la traducción fue realizada en Colombia, es factible el uso en otros países de habla hispana, puesto que, durante el proceso de traducción y adaptación cultural, se tuvo en cuenta que la versión ﬁnal en idioma español fuera el reﬂejo de la versión original en idioma inglés, y que fuera de fácil comprensión, evitándose el uso de términos ambiguos o modismos locales ^20^^,^^21^.

## Conclusiones

El proceso de traducción, adaptación cultural y confiabilidad del Perme Score y de la IMS fue realizado con una metodología rigurosa y se dispone de ambas escalas en idioma español, con excelente confiabilidad inter evaluador, en dos momentos diferentes (24 horas al ingreso/egreso de UCI). Se recomienda el uso de ambas herramientas en el contexto de la atención del paciente crítico para orientar y cuantificar las intervenciones de MT.
